# Multi-Level Attention Recognition of EEG Based on Feature Selection

**DOI:** 10.3390/ijerph20043487

**Published:** 2023-02-16

**Authors:** Xin Xu, Xu Nie, Jiaxin Zhang, Tingting Xu

**Affiliations:** School of Communication and Information Engineering, Nanjing University of Posts and Telecommunications, No. 66, XinMofan Road, Gulou District, Nanjing 210003, China

**Keywords:** EEG, SVM, multi-level attention, feature selection

## Abstract

In view of the fact that current attention-recognition studies are mostly single-level-based, this paper proposes a multi-level attention-recognition method based on feature selection. Four experimental scenarios are designed to induce high, medium, low, and non-externally directed attention states. A total of 10 features are extracted from 10 electroencephalogram (EEG) channels, respectively, including time-domain measurements, sample entropy, and frequency band energy ratios. Based on all extracted features, an 88.7% recognition accuracy is achieved when classifying the four different attention states using the support vector machine (SVM) classifier. Afterwards, the sequence-forward-selection method is employed to select the optimal feature subset with high discriminating power from the original feature set. Experimental results show that the classification accuracy can be improved to 94.1% using the filtered feature subsets. In addition, the average recognition accuracy based on single subject classification is improved from 90.03% to 92.00%. The promising results indicate the effectiveness of feature selection in improving the performance of multi-level attention-recognition tasks.

## 1. Introduction

Attention is the direction and concentration of psychological activities on an object, and is a psychological feature that accompanies mental processes, such as memory, thinking and imagination. The applications of attention-level recognition serve our lives in healthcare [1,2], safe driving [3,4], and education [5]. Posner divides attention into endogenous attention and exogenous attention [6]. Endogenous attention, also known as active attention, refers to the individual’s allocation of attention according to their goals or intentions to dominate behavior; exogenous attention, also knowns as passive attention, refers to the individual’s attention caused by external information, usually from unexpected stimuli. In the experimental paradigm designed by Posner and his colleagues, endogenous attention is aroused by presenting target cues in the fixation area, while exogenous attention is aroused by cues emerging in the vicinity of the target [7]. In this study, we focus on positive attention due to its importance in our daily life. We give the subjects clear goal intention through specific tasks, and induce the subjects’ attention state to varying degrees by controlling the difficulty of task execution.

In previous studies, attention levels can be identified by external representations, such as eye state and facial expression [8,9]. However, relying on external representations to identify attention may not be reliable [10]. With the development of cognitive psychology, researchers have found that the cerebral cortex is the most advanced area for generating attention. Attention reliably modulates neural activity in primary and secondary cortices, affecting the mean neuronal firing rate as well as its variability and correlation across neurons [11,12]. Therefore, attention-level recognition based on electroencephalogram (EEG) signals is gradually emerging.

EEG is a physiological signal produced by nerve activity of the brain, which can be obtained by placing electrodes on the surface of the human scalp. The neural activity of the brain changes with people’s mental state, emotion and cognitive activity [13,14]. Troy et al. recorded EEG signals during reading, painting, and other cognitive tasks in eight children with attention deficit disorder and eight normal children. The results showed significant θ band (4–8 Hz) amplitude difference between groups [15]. Ryota Kobayashi et al. collected EEG data from 61 healthy college students at rest with their eyes closed and concluded that individuals had higher attentional control at lower θ/β levels [16]. Li et al. acquired the EEG signals under three conditions of attention task, inattentive task and rest task through “tennis test” and “walking test”, and classified them by approximate entropy, sample entropy and multi-scale entropy features. The highest accuracy of 85.24% was obtained using sample entropy [17]. F Fahimi et al. developed an end-to-end depth Convolutional Neural Network (CNN) to decode attention information from EEG time series. Three different EEG representations were fed into the network, and the final average classification accuracy was 79.26% [18]. Hu et al. obtained three types of attention data through self-evaluation after online learning and extracted 25 features from 6 EEG channels, respectively. Using the correlation-based feature-selection (CFS) method and the K-nearest neighbor (KNN) classifier, an accuracy of 80.84% was reported in distinguishing the three attention states [19].

There are relatively few studies on multi-level attention classification based on EEG, and feature screening is rarely considered. An effective feature-selection algorithm can provide insights into the data, improve model generalization performance, as well as identify irrelevant features [20]. In this study, we choose a limited number of ten channels and filter the number of features, which reduce the computational complexity for the implementation of a miniaturized and intelligent detection device [21,22]. At the same time, multi-level attention recognition has more application prospects than single-level attention recognition. The four-level classification of attention avoids simple judgments and provides a transition interval for classifying attention states. This is practical, for example, in the process of detecting the driver’s attention and providing feedback when a downward trend in attention is detected (from high to medium), rather than until a low attention span occurs [23].

In this paper, four different experimental situations are designed to enable the subjects to achieve four states of high, medium, low and non-externally directed attention. A total of 10 features are extracted from EEG signals, including time-domain measurements, sample entropy, and frequency band energy ratios. Based on these features, an average recognition accuracy of 88.7% is achieved in classifying the four attention states using a support vector machine (SVM) classifier [24]. To further improve the classification performance and reduce the dimension of the feature space, feature selection is performed to identify the most informative features from the original feature set. In this work, we use the sequential-forward-selection (SFS) method [25] to generate the candidate feature subsets. Based on the optimal feature set, an improved classification accuracy of 94.1% was achieved, which demonstrates the effectiveness of the proposed feature selection and classification scheme in multi-level attention recognition.

The rest of this paper is organized as follows. In Section 2, we provide a detailed description of the experimental design. Section 3 explains the data processing procedures, including EEG preprocessing, data segmentation, and feature extraction. In Section 3, we show the classification results with different feature-selection methods. Section 4 and Section 5 are the discussions and conclusions, respectively.

## 2. Experimental Design

### 2.1. Channel Selection

EEG can be divided into five rhythms (frequency bands) of δ, θ, α, β and γ in the frequency domain, and different rhythms have different characteristics [26]. Among them, the frequency bands related to attention mainly include θ, α, and β waves, which have the following characteristics:

θ wave, with frequency ranging from 4 Hz to 8 Hz and amplitude ranging from 20 uV to 40 uV, usually occurs when people are relaxed or tired, and is mainly distributed in the central area of the brain. θ wave in awake state is related to attention alertness.

A wave, with frequency ranging from 8 Hz to 13 Hz and amplitude ranging from 10 uV to 80 uV, usually appears when people are calm, and is mainly distributed in the occipital and the parietal lobes.

B wave, with frequency ranging from 13 Hz to 30 Hz and amplitude ranging from 3 uV to 50 uV, usually appears when people are excited, and is mainly distributed in the frontal and the central areas.

According to previous research, compared with the non-attention state, EEG signals in the attention state have more β waves, but less θ waves and α waves [27]. Therefore, when selecting channels, we choose the ones in the frontal lobe, the central area (β wave), the occipital lobe, the parietal lobe (α wave) and the central area (θ wave). The final selected channels are Fp_1, Fp_2, F_3, F_4, C_3, C_4, P_3, P_4, O_1 and O_2.

### 2.2. Data Collection

14 subjects aged between 20 and 24 years old participated in this study, including 6 female students and 8 male students. All subjects were undergraduates or postgraduates, with right handedness and normal or corrected vision. Sufficient sleep was guaranteed before the experiment. The experiment was conducted in the Laboratory of Geography and Biology at Nanjing University of Posts and Telecommunications.

The laboratory has sufficient light and suitable temperature, which can make the subjects in a relaxed and comfortable atmosphere. The sound insulation effect is good, preventing uncontrollable factors outside the laboratory from interfering with the data acquisition process. During the experiment, electronic devices such as mobile phones are turned off to avoid electromagnetic interference generated by devices in the environment. The device used in the experiment is a multi-channel wet electrode EEG acquisition instrument produced by Nanjing Weisi Medical Institution. The experimental instrument can complete multi-channel EEG signal acquisition, amplification, sampling, filtering, etc. The electrode distribution conforms to the international 10-20 system standard electrode placement method.

### 2.3. Experimental Scheme

In contrast to the conventional two-level attention experiment, this experiment induced four different levels of attention states by controlling the difficulty of the tasks. The original data which met the experimental requirements based on self-evaluation scale were kept for further analysis. The four types of attention tasks are shown in Table 1.

### 2.4. Experimental Process

Before performing Task 1, the subjects were asked to do a set of numerical exercises. The experimenter named a number within 100. If the number was a prime number, the subjects were asked to answer “Yes”. If the number was not a prime number, the subjects were asked to say a factor of the number. For example, if the experimenter said “35”, the subjects could answer “5” or “7”. If the experimenter said “17”, the subjects should answer “Yes”. The purpose of setting the number exercise is to awaken the subjects’ sensitivity to numbers before starting the formal experiment.

The four types of tasks were carried out sequentially. The task time of Task 1 started when the subjects browsed the first number and ended when the last number was judged. The task time of Task 2 to Task 4 is the same as that of Task 1. After each task, the subjects rested for 30 s. During rest, they were asked to fill out corresponding questionnaires to self-evaluate their attention state during the experiment [28], so that we could screen the samples with subjective evaluations.

To ensure that the subjects were successfully induced to an appropriate attention state during the experiment, a subjective questionnaire was designed. At the end of each task, subjects were asked to fill out a corresponding questionnaire to assess the state they had just experienced during the experiment. The questionnaire for each task consisted of 3 questions, each with 5 options A–E. Each option received an increasing score from A, with 1 point for A and 5 points for E.

Two attention scales are shown in Table 2 and Table 3, respectively. For Table 2, a total score greater than 12 is considered to meet the expectations of Task 1, and 9–12 is considered to meet the expectations of Task 2. For Table 3, a total score of more than 12 is considered to meet the expectations of Task 3, and 9–12 is considered to meet the expectations of Task 4.

**Figure 1:**
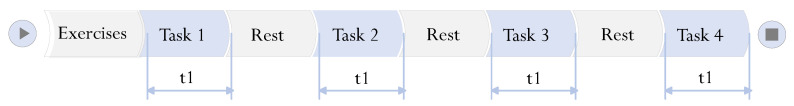
Flow chart of each group of experiments.

The purpose of the first question in Table 2 is to make sure that the subjects do not reject text tasks and digital tasks. During the experiment, all subjects were able to complete both the text tasks and the digital tasks (Score 5). The flow chart of each group of experiments is shown in Figure 1, where t1 is the time taken by the subjects to complete Task 1. At the end of the experiment, the experimental content was compared with the subjective scale scores, and the EEG signals that matched the purpose of the experiment were reserved for analysis, while those failed to match were invalidated. Each subject repeated the experiment in two groups with a 30-min interval between the two groups.

## 3. Data Processing

### 3.1. Data Preprocessing

**Figure 2:**
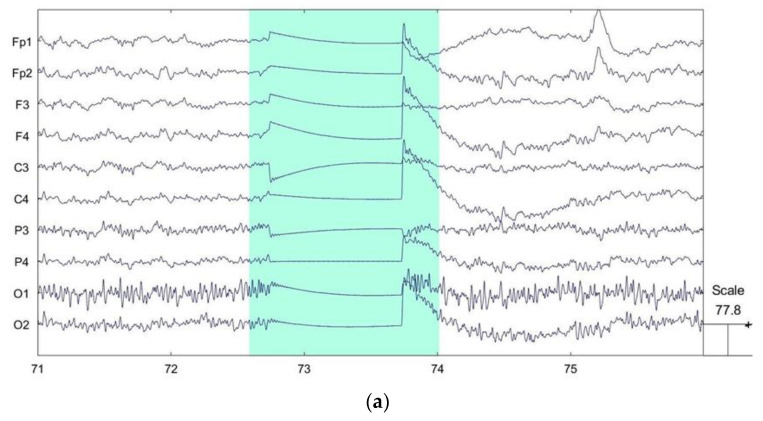

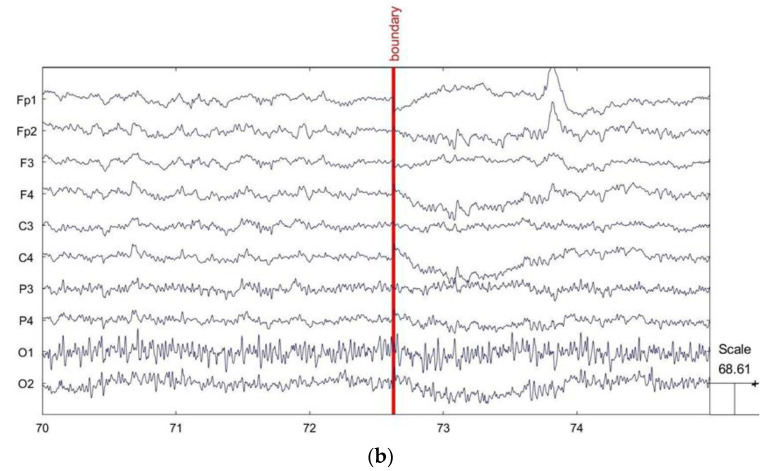
(**a**) The waveform of the EEG segment in the marked time interval is significantly different from the normal EEG signal outside the interval, which is an obvious bad segment. (**b**) EEG waveform with bad segments removed.

**Figure 3:**
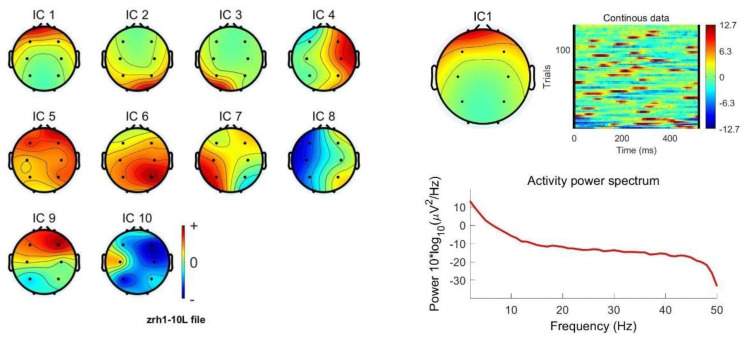
EEG topography of different signal components and attributes of electrooculogram artifacts. The energy distribution of the component containing electrooculogram artifacts (IC1) is concentrated in the forehead and can be identified and removed based on this characteristic.

**Figure 4:**
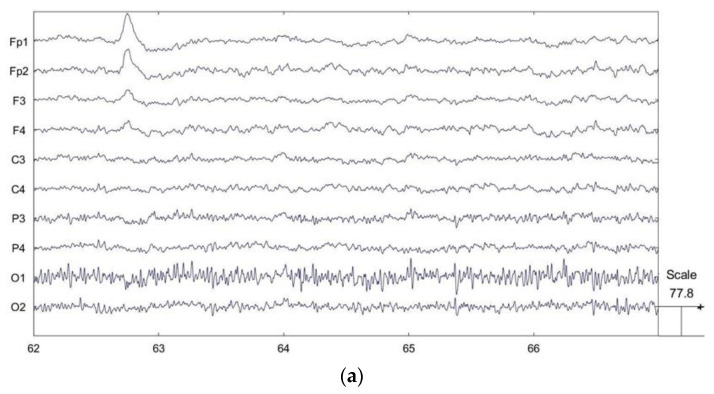

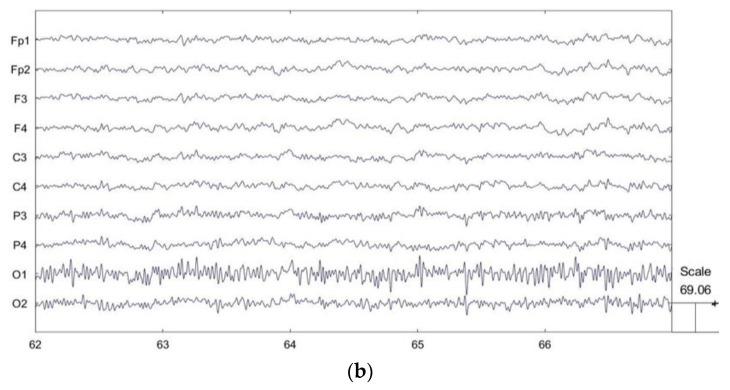
(**a**) EEG signal waveform fragments before preprocessing. There are obvious electrooculogram artifacts at around 63 s. (**b**) EEG signal waveform fragments after preprocessing. The eye movement artifacts are removed and the signal waveforms are stable and smooth.

EEG signals are susceptible to interference from environmental noise and other physiological signals such as eye electricity, myoelectricity and ECG during the acquisition process, and these interfering signals are mixed with EEG signals. To improve the signal-to-noise ratio of the EEG signal, the original signal needs to be preprocessed before feature extraction. The preprocessing in this experiment was performed using the EEGLAB toolkit in Matlab. In the experiment, the sampling rate was set to 512 Hz. The electrodes made good contact during the experiment, the impedance was below 10 kΩ, and there were no bad channels. First, the original signal was band-pass filtered using an FIR filter, preserving the signals in the frequency range of 0.5–30 Hz. The purpose is to remove noisy signals at higher frequencies such as power line interference. Obvious bad sections in the waveform can be manually selected and removed. Figure 2 shows the before and after comparison of bad segment removal. Finally, the low-frequency interferences such as eye electricity, myoelectricity and ECG are removed from the original signal using independent component analysis (ICA). ICA is a linear transformation with the main idea of separating signals into linear combinations of statistically independent non-Gaussian sources by minimizing the mutual information between the output components according to the InfoMax principle [29,30]. After performing ICA, artifacts such as oculoelectricity and myoelectricity in the components are manually identified and removed [31]. Figure 3 shows the EEG topography with different signal components and the main oculogram artifacts. Figure 4 shows the signal waveforms fragments before and after preprocessing. The artifact components are significantly reduced and the waveform becomes smooth.

### 3.2. Data Segmentation

**Figure 5:**
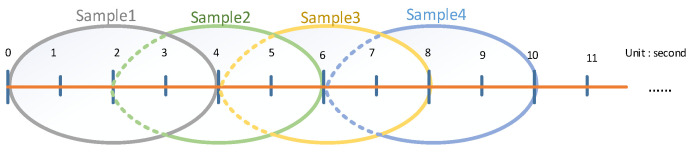
Schematic diagram of signal segmentation.

4-s time window with 2-s overlap is selected to segment the preprocessed EEG signals which allows each sample to contain over 2000 data points. The schematic diagram of the segmentation is shown in Figure 5 and the sample sources are shown in Table 4.

It can be seen from the above table that the number of samples from the four different attention levels are all between 800 and 900. The maximum ratio of different types of samples is 1.09, which makes it a balanced data set for multi-class classification task.

### 3.3. Feature Extraction

The purpose of feature extraction is to identify features from EEG signals that can reflect different attention states. Feature extraction methods can be divided into time domain, frequency domain and time-frequency domain analysis methods according to the types of feature parameters. The algorithms used for feature extraction can be divided into linear and nonlinear analysis methods. The time-domain features of EEG have the advantages of being specific, visual and easy to obtain, and therefore have been widely used by researchers [32,33]. Examples of time-domain features include mean, variance, peak value, peak-to-valley distance, fractal dimension, high-order zero-crossing analysis, etc. Frequency domain parameters include energy and power spectrum. The main methods are fast Fourier transform, autoregressive model, eigenvector, high-order spectrum and so on. Time-frequency domain analysis mainly employs wavelet transform to separate EEG signals into different rhythms, and use the root mean square of wavelet coefficients and energy as features for classification.

In this paper, the extracted features mainly include time-domain parameters (rectified mean value, maximum value, peak difference, root mean square, standard deviation and margin factor), sample entropy, and energy ratio (E_θ/E_all, E_α/E_all, E_β/E_all). These features are widely used and have shown great performance in attention recognition and other related EEG classification studies [34,35,36].

#### 3.3.1. Time-Domain Parameters

The following six time-domain features are selected. For N-point sequence $x\left(i\right)$:

1. Rectified average value: average value of absolute value of signals
  $$F1=\frac{1}{N}\overset{N}{\underset{i=1}{\sum }}\left|x\left(i\right)\right|$$
2. Maximum value
  $$F2=max\left(x\left(i\right)\right)$$
3. Peak difference: the difference between the maximum and minimum values of signals
  $$F3=max\left(x\left(i\right)\right)-min\left(x\left(i\right)\right)$$
4. Root mean square: the effective value of the signal
  $$F4=\sqrt{\frac{1}{N}\overset{N}{\underset{i=1}{\sum }}x{\left(i\right)}^{2}}$$
5. Standard deviation: the arithmetic square root of variance, which describes the degree to which a set of data is dispersed from the average value
  $$F5=\sqrt{\frac{1}{N}\overset{N}{\underset{i=1}{\sum }}{\left(x\left(i\right)-\bar{x}\right)}^{2}}$$
6. Margin factor: ratio of signal peak value to square root amplitude.
  $$F6=\frac{max\left|x\left(i\right)\right|}{\sqrt{\frac{1}{N}\sum_{i=1}^{N}x{\left(i\right)}^{2}}}$$

#### 3.3.2. Sample Entropy

Sample Entropy (SE) is a nonlinear measure of the complexity of a sequence [37]. It can be used to analyze mixed signals formed by superposition of deterministic signals and stochastic signals. Therefore, sample entropy is more suitable for EEG analysis than approximate entropy [38]. The algorithm is described as follows:

Construct m-dimensional vector $Y_{m}\left(i\right)$ in sequence by *N*-point sequence $X\left(i\right),\;\{Y_{m}\left(i\right),i=1,2,\ldots ,N-m+1$}.

$$Y_{m}\left(i\right)=\left\{x\left(i\right),x\left(i+1\right),x\left(i+2\right),\ldots ,x\left(i+m-1\right)\right\}$$

For each value of i, calculate the distance between the vector and other vectors, and the maximum distance $D\left\{Y_{m}\left(i\right),Y_{m}\left(j\right)\right\}$ is shown in the following formula:

$$D\left\{Y_{m}\left(i\right),Y_{m}\left(j\right)\right\}=max\left\{\left|x\left(i+k\right)-x\left(j+k\right)\right|,k=0,1,2,\ldots ,m-1.\;\;1\leq i,j\leq N-m,i\neq j\right\}$$

Calculating the proportion of the number satisfying the condition of $D\left\{Y_{m}\left(i\right),Y_{m}\left(j\right)\right\}<r$, ($N^{m}\left(i\right)$) to the total number $\left(N-m+1\right)$ from a given threshold r and a dimension m:

$$C_{i}^{m}\left(r\right)=N^{m}\left(i\right)/\left(N-m+1\right)$$

Average all $C_{i}^{m}\left(r\right)$:

$$B^{m}\left(r\right)=\frac{1}{N-m}\overset{N-m}{\underset{i=1}{\sum }}C_{i}^{m}\left(r\right)$$

Changing the dimension to m+1, and repeating the above steps to obtain $B^{m+1}\left(r\right)$, the sample entropy can be defined as

$$\mathrm{SampEn}\left(m,r\right)=-ln\frac{B^{m+1}\left(r\right)}{B^{m}\left(r\right)}$$

#### 3.3.3. Frequency Band Energy Ratio

**Figure 6:**
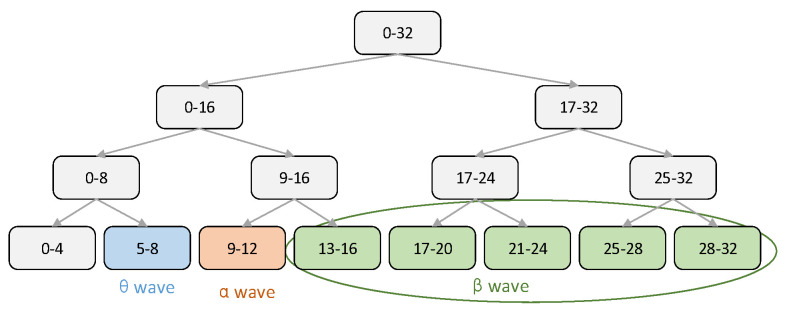
Schematic diagram of wavelet packet decomposition.

In this experiment, θ, α and β waves are separated from the original signal by wavelet packet decomposition [39]. EEG signals are non-stationary signals, and wavelet analysis and wavelet packet analysis are suitable for non-stationary signals. Wavelet analysis only further decomposes the low-frequency part of the signals, thus cannot well represent signals containing a large amount of detailed information. Compared with wavelet analysis, wavelet packet analysis can decompose both the low-frequency part and the high-frequency part, which makes the signal analysis more detailed and the time-frequency plane more detailed [40]. Therefore, in this paper, θ, α and β waves are separated from the original signal by wavelet packet analysis. The diagram of wavelet packet decomposition is shown in Figure 6.

By summing the squares of wavelet coefficients in different frequency bands, the energy of the corresponding frequency bands can be obtained, based on which the ratio of θ wave, α wave and β wave to the total energy of signals can be calculated.

**Figure 7:**
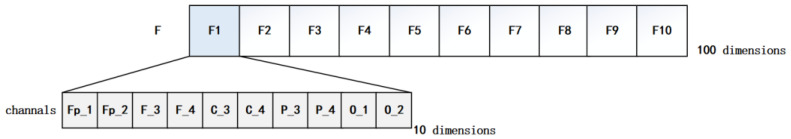
Illustration of feature vector F.

The final feature vector F is shown in Figure 7. F1 to F10 are rectified average, maximum, peak difference, root mean square, standard deviation, margin factor, sample entropy, E_θ/E_all, E_α/E_all, E_β/E_all, respectively.

## 4. Results

### 4.1. Classification

In this study, we employed support vector machine (SVM) to classify EEG signals from different attention states. SVM maps training examples to points in space to maximize the margin between classes. When the samples are not linearly separable, a kernel function can be used to map the samples in a low-dimensional space to a high-dimensional space for classification [41].

**Figure 8:**
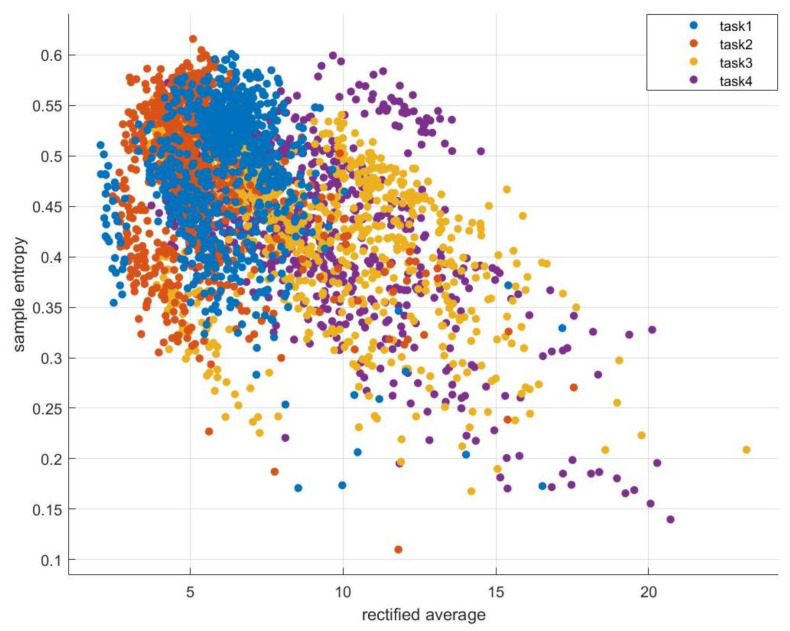
Scatter plot of the distribution of data points based on rectified average and sample entropy features.

We combined the samples from all subjects for classification training, which can expand the data set and verify the applicability of classification results to different individuals. From 3403 100-dimensional samples F, 60% were randomly selected as training set, 20% as validation set and 20% as test set. An accuracy of 88.7% was achieved using all 10 features for classification. Scatterplot of the distribution of data points based on rectified average and sample entropy features is shown in Figure 8.

To understand the importance of each feature in the classification task, and obtain the optimal feature combination, each of the 10 features is used for classification separately. The corresponding classification accuracy on the validation set associated with each feature are shown in Table 5.

From Table 5, the classification performance of some features is significantly better than that of the other features. To identify features that can effectively distinguish different states, feature screening is needed. Feature selection is a process of selecting some of the most effective features from a group of features to reduce the dimension of feature space [42].

### 4.2. Comparison of Different Feature-Selection Methods

Feature-selection methods can be divided into three categories: filter, wrapper, and embedded.

**Figure 9:**
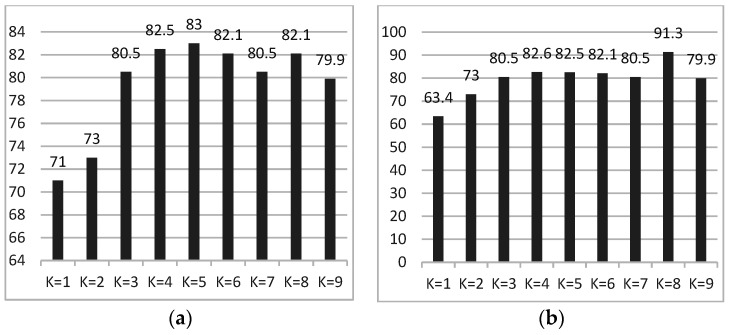
Accuracy under different K values. (**a**) Chi-square test; (**b**) Mutual information.

1. Filter, which scores each feature according to divergence or correlation, and trains the classifier by the selected feature subset [43]. Commonly used filter methods include the Chi-square test, mutual information method, variance selection method, correlation coefficient method and so on.
  The Chi-square test is to test the correlation between qualitative independent variables and qualitative dependent variables. In this paper, the SelectKBest function in feature_selection library is combined with the Chi-square test. By setting the value of parameter k, k best-performing features are selected, and the ranking of F1–F10 is F3 > F2 > F5 > F4 > F1 > F6 > F9 > F10 > F8 > F7. The classification performance of feature subsets with different k values is shown in Figure 9a. The highest classification accuracy of 83% is achieved on the validation set when K = 5, i.e., F1–F5 are selected as feature subsets.
  Similarly, the mutual information can also reflect the correlation between each feature and the tag. The larger the mutual information value, the stronger the correlation between the features and the tags. The ranking of F1–F10 is F2 > F3 > F1 > F5 > F6 > F4 > F9 > F7 > F10 > F8. The classification rates of feature subsets obtained when k takes different values are shown in Figure 9b. It can be seen from the figure that when K = 8, the highest classification rate in the validation set is 91.3%.
2. Wrapper: the wrapping method directly uses the performance of the classifier as the evaluation criterion, and selects multiple features at a time.
  Set the final optimal feature set as S. The 10 features were ranked according to the classification rate from high to low, as follows: F7 > F5 > F4 > F1 > F6 > F3 > F2 > F10 > F9 > F8. The above features are put into S in turn. If the accuracy is improved, keep the features, otherwise, discard them. The final feature subset (F7, F5, F4, F1, F6) achieved the highest classification rate of 94.5% in the verification set.
3. Embedded: Embedded methods integrate the feature-selection process with the classifier training process and mainly include penalty term-based feature-selection methods and tree-model-based feature-selection methods. Finally, F3, F6 and F7 were selected as feature subsets, and the classification accuracy in the verification set reached 93.2%

### 4.3. Classification Based on Wrapper Method

Based on the above analysis, the wrapper method is finally used for feature selection. More specifically, the sequential-forward-selection (SFS) method is employed to generate candidate feature subsets, which is a “bottom-up” search method. First, the target feature set is initialized as an empty set, and one feature is added to the feature set at a time. When the requirements are met, the obtained feature set is used as the feature-selection result. Using the wrapper method as the evaluation criterion, the learner is directly trained with the selected feature subset in the feature screening process, and the feature subset is evaluated according to the performance on the validation set.

The specific feature-selection steps are described as follows: setting the final optimal feature set as S; The 10 features were ranked according to the classification rate from high to low, as follows: F7 > F5 > F4 > F1 > F6 > F3 > F2 > F10 > F9 > F8. First, put F7 into S and then add F5; if classification accuracy improves after adding F5, keep F5 and update S; otherwise, delete F5; All remaining features are considered in turn according to the same principle, and the optimal feature set S of the original 10 features is finally obtained. The detailed steps for updating S are shown in Table 6.

From Table 6, the optimal feature set S should contain F7, F5, F4, F1, F6, i.e., sample entropy, standard deviation, root mean square, rectified average value and margin factor. Classification accuracy based on the optimal 50-dimensional feature vector is 94.5%.

Based on the results of the feature selection above, F was updated to give S, which contains 3403 50-dimensional samples. The model is trained on the updated training set, and the classification accuracy on the test set is improved to 94.1%, which is 5.4% higher than that before feature selection.

To verify the adaptability of feature selection between different subjects, we performed subject-specific classification for each subject using the two groups of data collected in different time periods. The first group of data was used as a training set, and the second group of data was used as a test set. The classification accuracy on the test set before and after feature selection are shown in Table 7.

As can be seen from Table 7, after feature selection, the classification accuracy of 11 out of 14 subjects has been improved. The average accuracy of 14 subjects increased from 90.0309% to 92.0026%. We used the S-W test to test the normality of the difference before and after feature extraction. *p* = 0.332 implies acceptance of the original hypothesis that the sample has normal distribution traits. To demonstrate the statistical significance of the feature screening results a paired t-test was performed as shown in Table 8. The results indicate the effectiveness of feature selection in improving classification performance.

## 5. Discussion

In this study, we classified the subjects’ attention state into four layers. After collecting the corresponding EEG signals, we extracted ten features including six time-domain features, three frequency domain features, and a nonlinear feature. These features are screened and optimized, and finally, the five best feature combinations are selected. Based on the optimal feature set, the classification accuracy is improved significantly compared with using the original feature set without feature selection.

**Figure 10:**
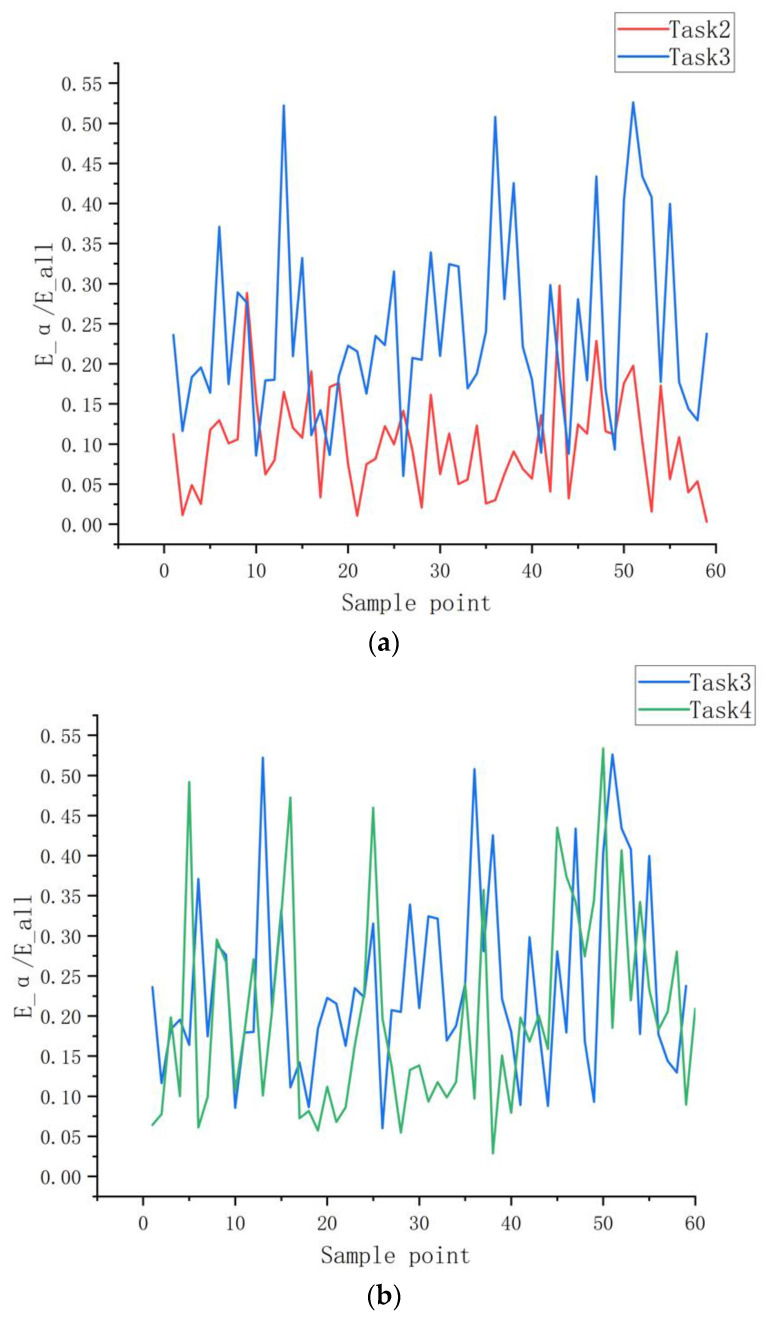
(**a**) E_α/E_all is significantly lower in medium attention task compared to low attention task. (**b**) Insignificant change in E_α/E_all in Low attention task vs. Non-externally directed task.

Experimental results show that feature F9 (E_α/E_all) was not included in the optimal feature set. In Figure 10a, we compare the F9 feature of Task 2 to Task 3 of the same subject. Significantly lower α power of medium attention task was observed compared with that of the low attention task, which is consistent with the previous findings [44,45,46]. However, for tasks 3 and 4, the α-wave energy ratio is indistinguishable, as shown in Figure 10b. This may have led to the exclusion of the α wave energy ratio from the optimal feature set.

The feature filtering algorithm in this paper can improve the classification performance while reducing the dimension of the feature set. The reduction of computation can also serve real-time EEG attention and fatigue detection system, which is promising [21,22]. In the current study, all 10 channels are included for feature extraction. Some studies have proposed a channel-based feature-selection method that takes into account the performance of a single-channel model and its physical location for studying groups of channels related to attention detection. This can be combined with feature screening to better improve classification performance [47].

In the feature-selection process, we tried three methods, and the results show that the combination of features obtained using the wrapper method achieves the highest accuracy. However, this does not prove that the wrapper method is superior to the other feature-selection methods. This is because the wrapper method enumerates all the different feature subsets and chooses the one that makes the model work best. This is suitable for this study when the number of features is small, but for applications with very large number of features, the time complexity of wrapper methods may be too high.

This study also has several limitations: (1) A time window of four seconds was chosen for data processing. Time windows of different lengths may have an impact on classification accuracy. (2) The number of subjects recruited and the range of their age was limited. Future studies should cover different age groups and expand the sample size. (3) The number of EEG channels could also be further screened to reduce the complexity of data processing [48].

In addition, the subjects in this experiment were all university students in normal physical and mental conditions. There are several diseases that can affect people’s attention levels, such as attention deficit hyperactivity disorder. Future studies will also be directed towards comparing people with attention deficits with healthy subjects [49].

## 6. Conclusions

In this paper, we designed four experimental scenarios to induce different levels of attention. Combined with the self-assessment questionnaire, the EEG signals of four states of high attention, middle attention, low attention and non-externally directed attention were collected.

After pretreatment, a total of 3403 samples from 14 subjects were obtained. Ten features are extracted from each of the ten EEG channels, which results in a 100-dimensional feature vector to classify the four categories of EEG signals. An 88.7% classification was achieved using a support vector machine classifier.

To identify the optimal subset of discriminating features from the original feature set, the sequence-forward-selection method is employed. After feature selection, sample entropy, standard deviation, root mean square, rectified mean value and margin factor are retained, based on which the classification accuracy was improved to 94.1%.

At the subject level, when using the first group of data for training and the second group of data for testing, the average classification accuracy was improved by 1.97% after feature selection. These promising results indicate the effectiveness of feature selection in attention-level recognition.

## Figures and Tables

**Table 1:** Four types of attention tasks.

	Attention Type	Task Content	Task Description
Task 1	High	Browsing and mental arithmetic	Browse a 10 × 10 number matrix (1–100 randomly distributed and not repeated) and find out the prime numbers.
Task 2	Medium	Browse	Browse the provided discussion text materials.
Task 3	Low	Distraction	Keep your eyes on the text in Task 2 and think about things unrelated to the task.
Task 4	non-externally directed	Rest	Try to relax and think nothing.

**Table 2:** Attention scales for Task 1 and Task 2.

Q	Do you not reject this task and could perform it completely?
A	A. Hard. B. Slightly difficult. C. General. D Relatively easy. E. Easy.
Q	Do you have a lack of concentration during the task due to nervousness, etc.?
A	A. Always. B. Often. C. No obvious perception. D. Occasionally. E. Almost never.
Q	Do you think you have achieved an ideal state of attention in the experiment?
A	A. Very unfocused. B. Less focused. C. Neutral. D. More focused. E. Very focused.

**Table 3:** Attention scales for Task 3 and Task 4.

Q	Do you have complex emotions such as tension and anxiety in the task?
A	A. Always. B. Often. C. No obvious perception. D. Occasionally. E. Almost never.
Q	Do you have a lack of concentration during the task due to nervousness, etc.?
A	A. Always. B. Often. C. No obvious perception. D. Occasionally. E. Almost never.
Q	Do you think you have achieved an ideal state of attention in the experiment?
A	A. Very unfocused. B. Less focused. C. Neutral. D. More focused. E. Very focused.

**Table 4:** Sample size of each subject under four types of tasks.

Num	Sample Number				Sum
	Task 1	Task 2	Task 3	Task 4	
1	32	59	59	74	224
2	59	65	62	62	248
3	71	69	69	69	278
4	55	59	59	60	233
5	53	59	59	59	230
6	46	52	49	50	197
7	60	62	59	59	240
8	54	62	60	61	237
9	80	77	79	79	315
10	65	63	69	71	268
11	66	66	63	69	264
12	48	49	49	49	195
13	51	59	51	57	218
14	65	63	64	64	256
Total	805	864	851	883	3403

**Table 5:** Classification accuracy of each feature on the validation set.

Sub-Vectors	Size	Feature	Classification Accuracy on the Validation Set
F1	3403 × 10	rectified average	83.2%
F2	3403 × 10	maximum	63.4%
F3	3403 × 10	peak difference	71%
F4	3403 × 10	root mean square	84%
F5	3403 × 10	standard deviation	84.4%
F6	3403 × 10	margin factor	81.65
F7	3403 × 10	sample entropy	84.8%
F8	3403 × 10	E_θ/E_all	32.1%
F9	3403 × 10	E_α/E_all	41%
F10	3403 × 10	E_β/E_all	43.6%

**Table 6:** Steps for updating the optimal feature set and the classification accuracy associated with each step.

Newly Added	Optimal Feature Set S	Size	Classification Accuracy	Retain or Not	Updated S
F7	F7	3403 × 10	84.8%	-	F7
F5	F7, F5	3403 × 20	93.2%	Yes	F7, F5
F4	F7, F5, F4	3403 × 30	93.7%	Yes	F7, F5, F4
F1	F7, F5, F4, F1	3403 × 40	94.2%	Yes	F7, F5, F4, F1
F6	F7, F5, F4, F1, F6	3403 × 50	94.5%	Yes	F7, F5, F4, F1, F6
F3	F7, F5, F4, F1, F6, F3	3403 × 60	94.3%	No	F7, F5, F4, F1, F6
F2	F7, F5, F4, F1, F6, F2	3403 × 60	93.6%	No	F7, F5, F4, F1, F6
F10	F7, F5, F4, F1, F6, F10	3403 × 60	90.2%	No	F7, F5, F4, F1, F6
F9	F7, F5, F4, F1, F6, F9	3403 × 60	91.6%	No	F7, F5, F4, F1, F6
F8	F7, F5, F4, F1, F6, F8	3403 × 60	89.5%	No	F7, F5, F4, F1, F6

**Table 7:** Comparison of classification accuracy before and after feature selection.

Num	Accuracy before Feature Selection	Accuracy after Feature Selection	Increase
1	79.2793	84.6847	+5.4054
2	87.8049	89.4309	+1.6260
3	90.7801	91.4894	+0.7093
4	87.2881	86.4407	−0.8474
5	89.7436	90.2655	+0.5219
6	92.9293	97.9798	+5.0505
7	94.2149	98.3471	+4.1322
8	89.8305	90.7563	+0.9258
9	85.2564	86.1635	+0.9071
10	89.7059	93.3824	+3.6765
11	95.4198	94.7368	−0.6830
12	94.9495	97.9798	+3.0303
13	91.8919	91.8919	0
14	91.3386	94.4882	+3.1496
average	90.0309 ± 4.26	92.0026 ± 4.45	1.9712 ± 2.08

**Table 8:** Result of paired *t*-test analysis. *p* = 0.004 indicates that the mean values of the two groups of data show significant differences.

		Average Value	Standard Deviation	Mean Value Difference	*t*	*p*
Pairs	after	92.0026	4.45	1.9712	3.546	0.004
	before	90.0309	4.26			

## Data Availability

The data presented in this study are available on request from the corresponding author. The data are not publicly available due to privacy restrictions.
